# Climate change and its differential impact on sexual and reproductive health and rights among women in Nepal

**DOI:** 10.3389/frph.2025.1603370

**Published:** 2025-08-22

**Authors:** Jagadishwor Ghimire, Khusbu Poudel, Kritee Lamichhane, Amit Timilsina, Madhav Prasad Dhakal, Parash Prasad Phuyal, Sumanjari Pradhan, Jivan Devkota, Sujan Karki

**Affiliations:** ^1^ Ipas Nepal, Kathmandu, Nepal; ^2^ University of Cyberjaya, Cyberjaya, Malaysia; ^3^ Institute for Population and Social Research, Mahidol University, Salaya, Thailand

**Keywords:** climate change, climate crisis, gender, sexual and reproductive health and rights, differential impact, Nepal

## Abstract

**Background:**

Nepal is highly affected by climate change, experiencing glacier melting, untimely rainfall, floods, landslides, forest fires, and droughts, which collectively impact over 10 million people. There is a larger impact of climate change on human health, but its impact on women's and girls’ sexual and reproductive health and rights is yet to be explored. Thus, this study aims to understand the linkages between climate change and the unique impact on gender and sexual, and reproductive health and rights (SRHR).

**Methodology:**

This is an exploratory cross-sectional study conducted using a mixed method in Kailali, Arghakhanchi, and Kapilvastu districts. A total of 384 women were selected using systematic random sampling from the upper, middle, and downstream of Khutiya and Banganga river basins. Focus group discussions and key informant interviews were conducted to capture their experiences. Descriptive, bivariate, and multivariate analyses were carried out for quantitative data using SPSS, and a thematic analysis for qualitative data.

**Results:**

The women who experienced two or more climate-included disasters were more likely to face gender-based violence (*P* < .05). The study also showed that women's autonomy in making decisions on Sexual and Reproductive Health and Rights has increased among women exposed to a higher number of climate-related risk (*P* < .001). Though more than 3/4th of women did not want more children, women who are exposed to more climate-related risks wanted more children (*P* < .001). The survey found that more than three-quarters (76.3%) of respondents knew about the legality of abortion, and 85% of respondents knew the place to go for abortion services. The result also revealed a significant reduction in sexual desire among women who were exposed to a higher number of climate events. These findings are also aligned with the qualitative information in the study.

**Conclusion:**

The findings demand strengthening the resilience of healthcare systems to withstand the impact of climate change, ensuring that essential sexual and reproductive health services, including abortion, contraception, and maternal healthcare, are available and accessible even during the climate crisis. The findings indicate the need for interventions that empower women, address gender-based violence, and integrate sexual and reproductive health into climate change adaptation in policies and programs.

## Introduction

Climate change, driven by direct or indirect human activity, alters the composition of the global atmosphere beyond the natural climate variability observed over time (1). According to the Intergovernmental Panel on Climate Change (IPCC) Fourth Assessment Report, the effects of climate change are expected to intensify, including an increase in the frequency and severity of extreme weather events (EWE). By 2,100, the global average surface temperatures are projected to rise by 1.1–6.4°C, leading to more frequent heat waves and episodes of heavy precipitation. The report also highlights the likely increase in precipitation at higher latitudes and the decrease in precipitation in most subtropical land areas (2).

Climate change has profound implications for various aspects of human health and impacts most heavily on low and middle-income countries (LMICs) with limited adaptive capacity, and also the vulnerable population of developed countries (3, 4). It is well-recognized that climate change disproportionately affects vulnerable populations globally (5, 6). Among the vulnerable population, women in South Asia (7) and low-income countries like Nepal are disproportionately impacted on their health and well-being as they are more vulnerable due to existing gender and social inequalities, and their role as primary caretakers of the family (5–8).

In Nepal, this vulnerability is acute as over 1.9 million people are highly vulnerable, with an additional 10 million at increased risk from climate change, affecting a larger section of the population in disaster incidents (9). Scientific studies have observed that Nepal is going through an increase in maximum temperature, mostly affecting the higher altitude regions (10, 11). These include changes in monsoon patterns, heatwaves, winter droughts, abnormal cold spells, decreased snowfall, rapid glacier melting, and heightened risks of floods, landslides, and glacier lake outburst floods (GLOFs) (2, 12). South Asia, particularly the Himalayan highlands, including the Tibetan plateau, as well as arid areas of Asia, is highly vulnerable to climate change, increasing the likelihood of EWE (13–15). Given that climate change is driving an increase in the frequency and intensity of EWEs, we examine the broader association of climate change and SRHR. Few studies conducted across the globe have highlighted that EWEs like floods and droughts often aggravate existing gender-based violence (GBV), as women become more vulnerable to sexual exploitation and trafficking in the aftermath of disasters (16–19). Additionally, adolescent girls and women bear a double burden due to their gender and age, placing them at increased risk of poverty, food insecurity, school dropout, transactional sex, sexual violence, sexually transmitted infections, early marriage, or early childbearing due to lack of access to and use of the prevention method (8, 20).

### Women

Sexual and Reproductive Health and Rights (SRHR) refers to the right of all individuals to make informed, autonomous decisions about their sexual and reproductive lives, free from coercion, discrimination, and violence (21, 22). It encompasses the right to access quality information, education, and services across nine core components: contraception, maternal and newborn health, safe abortion care (where legal), prevention and treatment of sexually transmitted infections (STIs), including HIV, comprehensive sexuality education (CSE), GBV prevention and response, infertility and subfertility care, sexual health, and reproductive cancers (21, 22). While SRHR includes all these areas, our study specifically focuses on selected components-namely, knowledge of abortion, experiences of GBV, and aspects of sexual desire as they relate to SRHR. Additionally, we examine bodily autonomy, focusing on the use of contraception, maternal and child health services, from a gendered perspective as well, recognizing its critical role in shaping individuals’ sexual and reproductive health outcomes. SRHR-related knowledge and practices have a direct impact on the health outcomes of women. Gaps exist between SRHR knowledge and practices, which leave them vulnerable to sexual ill health (23). The utilization of SRHR services by women is strongly influenced by their exposure to key factors such as education, family background, economic status, occupation, and exposure to media. These elements shape awareness, access, and attitudes toward SRHR. There is a significant link between a woman's autonomy in decision-making and her ability to exercise her sexual and reproductive health and rights effectively (24).

Besides growing threats of climate change to health worldwide, one critical but often overlooked dimension is its direct and indirect impact on SRHR, and the vulnerability is increased due to environmental and climate-related challenges (25). The recent study conducted by Ipas in Mozambique has also shown that climate change affects women's health by limiting their economic opportunities, worsening existing GBV, and interrupting access to SRHR services (26). Disruptions to health infrastructure due to climate events can limit access to essential services like prenatal care, contraception, emergency obstetric care, and GBV prevention and response services, which are critical for safeguarding women's reproductive rights and preventing unintended pregnancies, maternal mortality, and other forms of complications (8, 27).

The recent reports from the IPCC also highlight the many ways in which gender intersects with other factors, including age, race, socioeconomic status, and sexuality, to influence people's experiences with climate change (28–30). Women are more vulnerable to the effects of climate change due to their social, economic, and cultural roles and responsibilities. The Safe Motherhood and Reproductive Health Rights Act of 2018 in Nepal guarantees women's reproductive rights, aligning with the fundamental rights outlined in the Constitution of Nepal (2072 BS) (31, 32). As a priority under the Public Health Act (2075) and the National Health Policy (2076), the act ensures that everyone can experience safe motherhood and make informed choices regarding their reproductive health, establishing these rights as basic human rights (33, 34). Despite progressive policies, women in Nepal still face increased risks to their sexual and reproductive health and rights during and after climate-related events, which are often neglected (35). The increasing trend of men emigrating in search of work (16). This is resulting in the overburdening of household and community chores for women. Women are primarily responsible for water collection, agricultural work, and caregiving, roles increasingly challenged by environmental stressors.

The existing evidence in Nepal shows that climate change may exacerbate the burden of non-communicable diseases (NCDs), including nutrition risk, vector-borne diseases, water-borne diseases, heat risk, and mental health issues (36). It also has a significant impact on sexual and reproductive health and rights (37), and gender equality in LMICs (38). Nepal, being a low-income country, and experienced EWE and natural disasters, which have also affected socio-economic and demographic patterns. However, the interlinkages between climate, gender, and SRHR have been overlooked in the broader climate change discourse (39). The study conducted in protracted crises/emergencies suggests dedicated attention and effective coordination to ensure emergency needs and reproductive rights for women (40). In this context, understanding the interlinkages of climate change with gender and SRHR is crucial for developing gender-sensitive adaptation strategies and protecting women's health and rights (41). Thus, this study aims to understand the differential impact of climate change on gender and SRHR and identify the potential inclusive adaptation strategies.

## Materials and methods

### Study design and study areas

This exploratory cross-sectional study was conducted in two river basins (Khutiya and Badganga rivers) from Kailali, Arghakhanchi, and Kapilvastu districts in Nepal. These river basins were selected based on consisting of all three streams (upper, mid, and down) in a short distance, which can give the characteristics of different river streams. Both rivers originated from the Mahabharat Range (High hills), passed through the Chure (youngest hill) and ended up flowing in the plain areas. Further, the Khutiya river basin represents the Far west of Nepal, and Badganga river represents the middle part of Nepal. The study tried to cover the different socioeconomic and geographic areas of Nepal. At least one vulnerable settlement was selected from each stream in the study areas. The study consists of a mixed-methods design, following both quantitative and qualitative methods.

### Sampling

In the first phase, households from the vulnerable settlements of upstream, mid, and downstream were listed from both river basins, as we took the river basin approach. Upstream is more prone to landslides, midstream midpoint for landslides and floods, however, downstream is more prone to floods. Eligible households and women aged between 18 and 49 were identified from the list. A single sampling frame was created for systematic random sampling based on census household and eligible women. The sampling frame for the study was women aged between 18 and 49 residing in the study areas. A total of 832 women who have been continuously residing in the study area for five years were eligible for the study, which was the final list for the sampling. Climate-related risk is the major dependent variable of this study. Based on systematic random sampling considering a 50 percent prevalence (p) of effects from climate-related risks for population (P) of 832 as there was no previous similar study to get the prevalence, design effect of 1 for systematic random sampling, 10 percent relative margin of error (e), and 90 percent response rate (r), 423 women were selected as a sample for the study using formula z^2^p(1-p)d/e­^2^rP. The sampled women were selected and identified through systematic random sampling from the eligible women's list.

For the qualitative data, in total 12 focused group discussions (FGD) with the women, and 22 key informant interviews (KII) with women and men from different backgrounds [four health facility incharges, six female community health volunteers (FCHVs), six local government representatives, and one member from six different community forest user groups (CFUGs) member who are the primary beneficiaries of community forest] to understand the perceived impact of climate change in gender, GBV and SRHR. The health facility in charge of all facilities of study area was interviewed. The FCHVs and elected members were selected from the respective wards and CFUGs members were selected as per the recommendations of CFUG. The FGDs were done to qualify the quantitative findings, and KII were carried out with key informants of the study areas to get broader information and context on the study. The research participants for the FGDs were selected purposively from two groups to understand the intergenerational experiences, first six from the women aged 18–49 years, and the second six groups of women aged more than 50 years. Similarly, KII participants were also identified and selected purposively. The participants for FGDs were selected as per the recommendations of CFUG. The sample size for both FGDs and KIIs was taken considering the saturation principle (42–44).

### Training and data collection

The 12 field data collectors were trained for two days on the research tool, questionnaire, KII, and FGD guidelines. The study team leader led the training and was assisted by subject experts. The questionnaires were pretested in a similar setting in a non-study area and revised based on pretest reports. After the finalization of the questionnaire, they were trained in data collection using KoboCollect. KoboCollect is an open-source android-based application used for mobile data collection, mostly designed for humanitarian action, development, environmental protection, research, and social good. This application is part of the KoBoToolbox data collection platform.The data collection was carried out through face-to-face interviews by trained interviewers. Few sampled women were unavailable in the study area for the interview during the field study period, which was covered by our oversampling for non-response rate.

### Measures of variables

To evaluate the associationof climate events, seven different dependent variables (facing GBV, women's autonomy for making decisions on SRHR, knowledge of legality of abortion, intention to have children, extra work at home, self-sexual desire, and partner's sexual desire) were used for the analysis. These variables were prioritized following the literature review, expert, and community consultation. While asking the respondents, the statement was read aloud which was “Please share your experience/opinion based on your experiences in latest climate event that you have faced”. For the major independent variable, exposure to climate-related risk was used. The climate-related risks for this study are floods, river cutting, and heat waves, which are more prevalent in the study areas. Exposed to at least two events among three was defined as more exposed, and one or no event was defined as less or no exposure. Since this is a cross-sectional study with limited prior evidence for this specific context, we compared the findings between two groups: those more exposed to climate-related events (experiencing two or more climate-related events) and those less or not exposed (one or no events). Exposure to climate-related risk is a key explanatory variable to assess whether such risks significantly affect the SRHR of women. The survey question for this variable was “What are the events (climate induced disasters) that you faced in the last 5 years?” and the responses were multiple which are flash floods, river cutting, heat wave, landslides and others.

The women's autonomy, intention to have children, and sexual desires are calculated for currently married women only. Other variables are calculated based on all women's samples. The six dependent variables are based on each specific question, but the women's autonomy variable is an index variable comprising three different variables (decide on health care for themselves, decide on the use or non-use of contraception, and can say no to sex with their husband/partner if they do not want to). Three questions were Who usually makes decisions about health care for yourself: you, your (husband/partner), you and your (husband/partner) jointly, or someone else? (a). Would you say that using contraception is mainly your decision, mainly your (husband's/partner's) decision, or did you both decide together? and (b). Can you say no to your (husband/partner) if you do not want to have sexual intercourse? The decision is considered autonomous if a woman makes it either independently or jointly with her husband. It is considered non-autonomous if the decision is made solely by her husband or another family member. If all three conditions are satisfied, such women are categorized as autonomous in decision-making of SRHR as outlined by Sustainable Development Goals (SDGs) 5: indicators 5.6.1 (45, 46). Existing literature suggests that unintended pregnancies often increase during and after climatic events, and most of the unintended pregnancies end with induced abortion. In this context, the study also assessed respondents' knowledge of abortion legality of abortion, which may help to search for safe abortion services. Women who responded that abortion is legal in Nepal were coded as one, while those who did not were coded as zero (Is abortion legal in Nepal) (47). Respondents who experienced at least one episode of any type of violence—whether—were also coded as one, and those who did not face any type of violence were coded as zero (Did you face any violence? If Yes, What were the types of violence during that time? And response category were multiple- physical, economic, psychological, or sexual. Similarly, women who performed extra work at home compared to normal were coded as one, while those who did not were coded as zero (Did you do extra work at home compared to normal time?). A similar dichotomization was applied to intentions to have children after a climatic event, with a response of one indicating intention and zero indicating none (Did you have desire of more children after climate events?). Additionally, women or their husbands who reported an increase or maintenance of sexual desire after the climatic event were coded as zero, whereas those who reported a decrease in sexual desire were coded as one [Was there changes in your sexual desire during climate event? Was there changes in your husband/partner sexual desire during climate event? The responses were no change (same), increase and decrease].

### Data processing and analysis

The data collected were downloaded from Kobo Collect and transferred to SPSS. The personal identity was deleted during data cleaning before data analysis to keep confidentiality. For KII and FGD, code numbers were given to each participant. The data were cleaned and analyzed in terms of descriptive, bivariate, and multivariate analyses using SPSS.

The qualitative information was analyzed using thematic analysis in Microsoft Excel. The interviews were conducted in Nepali and local language Tharu, recorded and subsequently transcribed into Nepali language and translated into English. Coding was performed by a single coder to maintain consistency in theme identification and interpretation. Following the coding process, related codes were grouped into broader themes and subthemes that reflected recurring patterns across the dataset. These themes were carefully reviewed and refined to ensure internal consistency and accurate representation of the participants' experiences and perspectives. Each theme was clearly defined and named to capture its core meaning. The final analysis involved interpreting the themes in light of the research objectives and contextual factors, using illustrative quotes to support key findings. The qualitative findings complement the quantitative findings of this study.

### Consent and ethical approval

The field researcher clearly described the objectives of the study and the broader context to help participants understand why they are participating in the interview. The details of the risks and benefits of participating in the study were briefed before giving consent. The written consent was taken from the respondents before starting the interviews. The ethical approval of the study was obtained in September 2022 from the Nepal Health Research Council (Approval ID: 412/2022 P).

## Results

During the field study, a total of 384 women aged between 18 and 49 years were interviewed. The demographic characteristics of census women of study areas and the sampled women were found to be similar (16). The study results revealed that across both river basins, most of the respondents (58.9%) are aged 30–49, with 45.1 percent having basic education, though a significant portion (30.5%) were illiterate or had non-formal education. Ethnically, the majority (33.6%) were Hill Janjati, followed by Tharu (27.6%), Brahmin/Chhetri (19.5%), and the least being Hill Dalit (19.3%), while 89.8% relied on agriculture for their livelihood (Table 1). The results (Table 1) showed that women were exposed to climate-related risk and adverse climate events in the study areas. Nearly half of the women (46.9%) were exposed to one or no climate-related risk, whereas more than half (53.1%) were exposed to at least two, reflecting considerable climate vulnerability across both river basins. While women between the ages of 18 and 29 were evenly distributed across both exposure groups, women between the ages of 30 and 49 (55.3%) were exposed to multiple risks. Education revealed that the majority of women with non-formal or no education were exposed to two or more climate-related risks (69.2%). Ethnic groups such as Tharu (89.6%) and Hill Dalits (62.2%) were significantly more vulnerable to climatic threats, while Brahmin/Chhetri (69.3%) and Hill Janjati (69.0%) were comparatively less exposed. Most women (53.3%) working in agriculture faced numerous risks, and those who lived downstream (72.2%) were far more vulnerable than those who lived upstream (5.7%). This demonstrates that women who are downstream, less educated, and members of marginalized ethnic groups are more likely to be exposed to climate-related risk.

**Table 1:** Women who are exposed to climate-related risk by different characteristics.

Background Characteristics	Total respondents		Exposed to climate-related risk	
	Total (N)	Percentage	Exposed to one or no climate-related risk	Exposed to at-least 2 climate-related risk
Age				
18–29 years	158	41.1	50.0	50.0
30–49	226	58.9	44.7	55.3
Education				
Basic	173	45.1	50.9	49.1
Secondary and higher	94	24.5	59.6	40.4
Nonformal and illiterate	117	30.5	30.8	69.2
Ethnicity				
Brahmin/Chhetri	75	19.5	69.3	30.7
Hill Janjati	129	33.6	69.0	31.0
Hill Dalit	74	19.3	37.8	62.2
Tharu	106	27.6	10.4	89.6
Occupation				
Agriculture	345	89.8	46.7	53.3
Other	39	10.2	48.7	51.3
River Stream				
Upstream	88	22.9	94.3	5.7
Midstream	116	30.2	40.5	59.5
Down stream	180	46.9	27.8	72.2
Total	384	100.0	46.9	53.1

### Climate-related risk and gender based violence

The findings of this cross-sectional study showed that there is a significant association of climate-related risks and GBV among women between the two climate-related risk groups. The Table 2 presents the women who have faced GBV based on various background characteristics and their climate-related risk exposure. Overall, 21.1 percent of the women had experienced GBV during adverse climate events. Those exposed to at least two climate-related risks significantly (*P* < .001) had faced GBV (30.9%) compared to those with one or no climate-related risk (10.0%). Women with non-formal or no education faced higher rates of GBV (34.2%). In contrast, those with secondary education experienced much lower rates (9.6%), indicating a statistically significant association between education and women facing GBV (*P* < .001). Ethnicity-wise, Tharu women were most affected (53.8%), while Brahmin/Chhetri (8.0%) and Hill Janjati (7.0%) women faced significantly less GBV (*P* < .001). Geographically, women living downstream (35.0%) were more vulnerable to GBV compared to those upstream (19.3%) or midstream (0.9%) (*P* < .001).

**Table 2:** Women who have faced GBV by different characteristics.

Background characteristics	The percentage of women faced the GBV			*χ*^2^ (*P* value)
	Not faced	Faced	Total (*N* = 384)	
Climate-related risk***				
Exposed to one or no climate-related risk	90.0	10.0	180	**χ^2^** = 20.053 *P* < .001
Exposed to at-least 2 climate-related risk	69.1	30.9	204	
Age				
18–29 years	79.7	20.3	158	**χ^2^** = 1.053 *P* = 0.350
30–49	78.3	21.7	226	
Education***				
Basic	81.5	18.5	173	**χ^2^** = 20.248 *P* < .001
Secondary and higher	90.4	9.6	94	
Nonformal and illiterate	65.8	34.2	117	
Ethnicity***				
Brahmin/Chhetri	92.0	8.0	75	**χ^2^** = 94.732 *P* < .001
Hill Janjati	93.0	7.0	129	
Hill Dalit	87.8	12.2	74	
Tharu	46.2	53.8	106	
Occupation				
Agriculture	78.6	21.4	345	**χ^2^** = 0.258 *P* = 0.685
Other	82.1	17.9	39	
River Stream***				
Upstream	80.7	19.3	88	**χ^2^** = 49.607 *P* < .001
Midstream	99.1	0.9	116	
Down stream	65.0	35.0	180	
Total	78.9	21.1	384	

*** *P* < 0.001.

Source: Field Survey, 2022.

The participants from FGD also shared their own experiences on GBV during the climate crisis. They said that the climate crisis affects them from all spheres of life, including emotional, social, and economic dimensions. As a result of the crisis, they lost everything they had with them. Women are even more at risk during this period.

“When the flood erodes away the cultivated land and crops, there is decreased production, resulting in conflict at home. Some abusers see the natural calamities as the opportunity to do sexual harassment as well”. FGD participant

### Climate-related risks and women's autonomy in decision-making for SRHR

The findings from Table 3 showed that slightly more than half the women have autonomy in decision-making for SRHR. Women's autonomy in making decisions on SRHR has increased among women exposed to a higher number of climate-related risks. Out of 359 women, those facing at least two climate-related risks exhibited significantly greater autonomy (62.2%) than those exposed to one or no climate-related risks (38.6%), with a highly significant difference (*P* = 0.0001). Tharu women (65.3%) had higher autonomy, whereas Brahmin (37.5%) had the lowest autonomy (*P* = 0.003). Upstream women responded having the lowest autonomy (32.1%) compared to midstream (57.1%) and downstream (56.4%) women (*P* < .001). Age and education showed no significant association with decision-making autonomy. As this finding suggests that more vulnerable women are autonomous, it seems contradictory; however, qualitative findings supported that after a disaster, temporary male migration for searching labor was increased, and all responsibilities were taken over by left-behind women. Further, while running seasonal migration variable in the same study found that the tendency of male migration is significantly increased among more exposed group than in the less or no exposed group (24% vs. 15%, data table not shown).

**Table 3:** Percentage of women who have autonomy in decision-making for SRHR by different characteristics.

Background Characteristics	Percentage of women's autonomy in decision-making for SRHR			χ^2^ (*P* value)
	No	Yes	Total (*N* = 359)	
Climate-related risk***				
Exposed to one or no climate-related risk	61.4	38.6	171	**χ^2^** = 94.732 *P* < .001
Exposed to at least 2 climate-related risk	37.8	62.2	188	
Age				
18–29 years	51.4	48.6	140	**χ^2^** = 0.531 *P* = 0.516
30–49	47.5	52.5	219	
Education				
Basic	51.8	48.2	168	**χ^2^** = 4.321 *P* = 0.115
Secondary and higher	54.3	45.7	81	
Nonformal and illiterate	40.9	59.1	110	
Ethnicity**				
Brahmin/Chhetri	62.5	37.5	72	**χ^2^** = 13.759 *P* = 0.003
Hill Janjati	52.1	47.9	121	
Hill Dalit	50.0	50.0	68	
Tharu	34.7	65.3	98	
Occupation				
Agriculture	48.6	51.4	329	**χ^2^** = 0.243 *P* = 0.704
Other	53.3	46.7	30	
River Stream***				
Upstream	67.9	32.1	84	**χ^2^** = 15.575 *P* < .001
Midstream	42.9	57.1	112	
Down stream	43.6	56.4	163	
Total	49.0	51.0	359	

** *P* *<* *0.01*.

*** *P* *<* *0.001*.

Source: Field Survey, 2022.

The focused group discussions' findings suggested that women prefer to have their male members in the family for more support during the crisis. This helped them to have a better decision during the difficult time, whether it is related to moving to a temporary shelter, or feeding their children or even for the use of SRHR services, antenatal, delivery or post-natal checks, contraception, or other services if they needed it during the period.

“Women request their male family members to be present in the decision-making roles during rescue. Men have more willpower, and if men are present during the time of disaster, they will have more confidence, and it will be easier to make decisions”. KII participant

### Climate-related risks and workload for women

The Table 4 showed the percentage of women who reported increased workload at home, where three out of five (60.9%) women reported that they have increased workload. Exposure to at least two climate-related risks showed a strong association with increased household work, with 75% reporting extra work, compared to 45 percent among those with one or no climate-related risk (*P* < .001). Educational disparities were also significant; women with nonformal or no education reported extra workload (76.9%), indicating a decreased workload among educated women (*P* < .001). Ethnicity was also statistically significant, with Tharu women reporting the highest extra work (83%) and Brahmin/Chhetri women with the least (34.7%) (*P* < .001). Downstream women significantly reported the highest burden (76.7%) as compared to higher streams (*P* < .001). Whereas age and occupation showed small differences and were not statistically associated with reported extra work.

**Table 4:** Percentage of women who worked extra work after climate events by different characteristics.

Background Characteristics	Percentage of women who reported that they have extra work at home			χ^2^ (*P* value)
	Not reported	Reported extra work	Total (*N* = 384)	
Climate-related risk***				
Exposed to one or no climate-related risk	55.0	45.0	180	**χ^2^** = 36.155 *P* < .001
Exposed to at least 2 climate-related risks	25.0	75.0	204	
Age				
18–29 years	43.0	57.0	158	**χ^2^** = 1.782 *P* = 0.182
30–49	36.3	63.7	226	
Education***				
Basic	45.1	54.9	173	**χ^2^** = 18.263 *P* < .001
Secondary and higher	47.9	52.1	94	
Nonformal and illiterate	23.1	76.9	117	
Ethnicity***				
Brahmin/Chhetri	65.3	34.7	75	**χ^2^** = 43.883 *P* < .001
Hill Janjati	41.9	58.1	129	
Hill Dalit	39.2	60.8	74	
Tharu	17.0	83.0	106	
Occupation				
Agriculture	39.4	60.6	345	**χ^2^** = 0.183 *P* = 0.669
Other	35.9	64.1	39	
River Stream***				
Upstream	61.4	38.6	88	**χ^2^** = 39.828 *P* < .001
Midstream	46.6	53.4	116	
Down stream	23.3	76.7	180	
Total	39.1	60.9	384	

*** *P* *<* *0.001*.

Source: Field Survey, 2022.

The qualitative research findings also reveal that women had increased workloads during the time of climate crisis. They gave examples of a longer period collecting drinking water as the usual sources were disturbed, especially in mid and downstream areas. It also increased other household chores for women.

“Workload for females has increased during times of natural calamities like flood. We have to be engaged in the household works, looking after children and cattle as usual along with additional tasks like managing firewood”. FGD Participants

### Climate-related risks and intention to have children

Only one in fourth women had the intention to have more children. The Table 5 showed that women exposed to at least two climate-related risks were more likely to intend to have more children (29.4%) compared to those exposed to one or none (16.7%), with a significant *P*-value of 0.005. Tharu women are the most likely (47.9%) to want more children whereas Hill Janjati women were least likely (*P* = 0.0001). Similarly, location by river stream was also significant, where downstream women (36.1%) were most likely to intend more children, followed by upstream (31.7%), whereas none from midstream intended to have more children (*P* = 0.0001). Other factors like age, education, and occupation had no significant differences.

**Table 5:** Percentage of women who have the intention to have more children after climate events by different characteristics.

Background Characteristics	Percentage of women who have intention to have more children after climatic event			χ^2^ (*P* value)
	No intention	Have intention	Total (*N* = 359)	
Climate-related risk**				
Exposed to one or no climate-related risk	83.3	16.7	162	**χ^2^** = 7.844 *P* = 0.005
Exposed to at least 2 climate-related risk	70.6	29.4	187	
Age				
18–29 years	73.2	26.8	138	**χ^2^** = 1.396 *P* = 0.248
30–49	78.7	21.3	211	
Education				
Basic	78.7	21.3	164	**χ^2^** = 1.101 *P* = 0.577
Secondary and higher	76.6	23.4	77	
Nonformal and illiterate	73.1	26.9	108	
Ethnicity***				
Brahmin/Chhetri	78.3	21.7	69	**χ^2^** = 48.611 *P* < .001
Hill Janjati	91.6	8.4	119	
Hill Dalit	83.1	16.9	65	
Tharu	52.1	47.9	96	
Occupation				
Agriculture	75.9	24.1	320	**χ^2^** = 0.688 *P* = 0.498
Other	82.8	17.2	29	
River Stream***				
Upstream	68.3	31.7	82	**χ^2^** = 51.235 *P* < .001
Midstream	100.0	0.0	112	
Downstream	63.9	36.1	155	
Total	76.5	23.5	349	

** *P* < 0·01.

*** *P* < 0·001.

Source: Field Survey, 2022.

The findings from the FGDs were mixed regarding the intention to have children in the areas affected by climate change. One group shared that it will be difficult to raise more children in such areas as it requires lots of effort to take care of children and other household chores. It is not only their decision, they also need to listen to their partners. In contrast, other groups of women shared that they want to have more children due to different reasons.

“We are in a remote area. If we have more children, they will earn more, loss of a child during the time of disaster can be compensated by other children at home”. FGD participants, above 50 years

### Climate-related risks and knowledge on abortion

The study tried to get the knowledge on abortion legality of the respondents in vulnerable areas which may help to search for safe abortion services. The result is to compare between more vulnerable and less. The results from Table 6 revealed that three out of four respondents (76.3%) know abortion legality. The majority of women with higher education (83.0%) knew about the legality of abortion (*P* < .001), while those with nonformal education or who were illiterate had the lowest level of knowledge (61.5%). Among ethnic groups, Hill Janjati women had the highest awareness (91.5%), whereas Tharu women had the lowest (49.1%) (*P* < .001). Geographically, all women living midstream were aware of abortion legality, while only 52.8 percent of downstream women had this knowledge (*P* < .001). Climate-related risk, age, and occupation did not show any significant differences in abortion awareness in the study area.

**Table 6:** Percentage of women who know about the legality of abortion by different characteristics.

Background characteristics	Percentage of women who know about the legality of abortion in Nepal			χ^2^ (*P* value)
	No knowledge	Knowledge on legal abortion	Total (*N* = 384)	
Climate-related risk				
Exposed to one or no climate-related risk	20.6	79.4	180	**χ^2^** = 0.174 *P* = 0.174
Exposed to at least 2 climate-related risk	26.5	73.5	204	
Age				
18–29 years	22.2	77.8	158	**χ^2^** = 0.355 *P* = 0.551
30–49	24.8	75.2	226	
Education***				
Basic	17.3	82.7	173	**χ^2^** = 20.287 *P* < .001
Secondary and higher	17.0	83.0	94	
Nonformal and illiterate	38.5	61.5	117	
Ethnicity***				
Brahmin/Chhetri	14.7	85.3	75	**χ^2^** = 63.799 *P* < .001
Hill Janjati	8.5	91.5	129	
Hill Dalit	20.3	79.7	74	
Tharu	50.9	49.1	106	
Occupation				
Agriculture	23.5	76.5	345	**χ^2^** = 0.091 *P* = 0.763
Other	25.6	74.4	39	
River Stream***				
Upstream	6.8	93.2	88	**χ^2^** = 104.982 *P* < .001
Midstream	0.0	100.0	116	
Down stream	47.2	52.8	180	
Total	23.7	76.3	384	

*** *P* *<* *0·001*.

Source: Field Survey, 2022.

**Figure 1:**
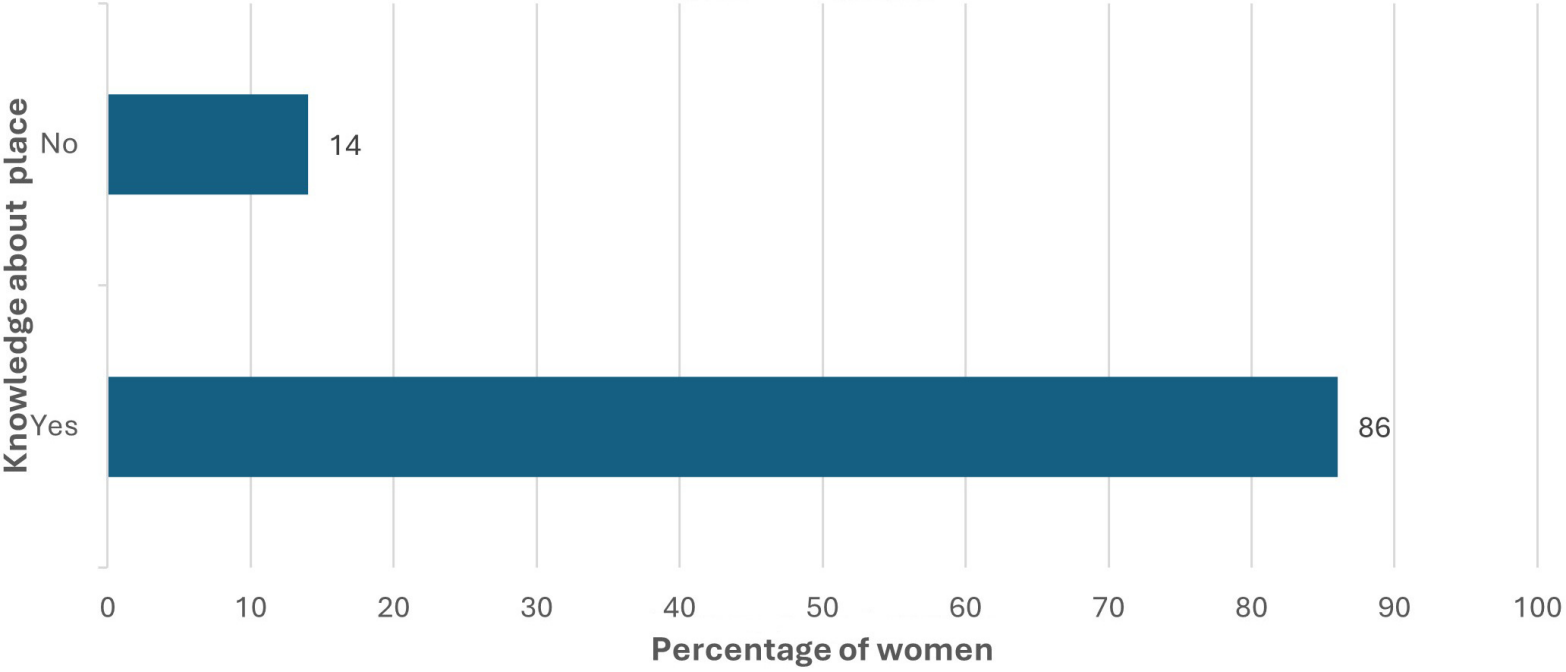
Know about the place to receive abortion services during crisis.

The women were also asked about their knowledge regarding the availability of SRHR services near to them to understand their preparedness if there are any difficulties. Most women in the study areas were aware (86%) of the place to access safe abortion services during the crisis, which indicated that women have the knowledge and can refer their family and friends if they need the services (Figure 1).

### Climate-related risks and sexual desire

The study asked about the sexual desire of women and their husbands during climate-related risk and whether the desire was the same, increased, or decreased. Few respondents (3% for self and 0.8% for partner) reported that increase in sexual desire during climatic events. About half of the respondents said that their desire was the same, and the rest said there was a decrease in sexual desire. Similar findings were reported about their husband/partner's sexual desire by women. Increased and same desire were merged together for the analysis.

Table 7 showed that sexual desire was significantly decreased among women who were exposed to at least 2 or more climate-related risks (*P* < .001). Similarly, higher aged women have less sexual desired compared to younger women in the survey areas. The education and caste/ethnicity of women also associated with decreasing in sexual desire. The upstream women have no difference in sexual desire compared with women resides in midstream and downstream.

**Table 7:** Percentage of women who reported their changes in sexual desire after climate events by different characteristics.

Background characteristics	Percentage of women who reported about changes in sexual desire			χ^2^ (*P* value)
	Same or increased	Decreased	Total (*N* = 359)	
Climate-related risk***				
Exposed to one or no climate-related risk	69.6	30.4	171	**χ^2^** = 58.331 *P* < .001
Exposed to at least 2 climate-related risk	29.3	70.7	188	
Age**				
18–29 years	56.4	43.6	140	**χ^2^** = 5.823 *P* = 0.016
30–49	43.4	56.6	219	
Education*				
Basic	48.8	51.2	168	**χ^2^** = 8.571 *P* = 0.014
Secondary and higher	60.5	39.5	81	
Nonformal and illiterate	39.1	60.9	110	
Ethnicity***				
Brahmin/Chhetri	84.7	15.3	72	**χ^2^** = 53.744 *P* < .001
Hill Janjati	38.8	61.2	121	
Hill Dalit	51.5	48.5	68	
Tharu	31.6	68.4	98	
Occupation				
Agriculture	47.1	52.9	329	**χ^2^** = 2.896 *P* = 0.089
Other	63.3	36.7	30	
River Stream***				
Upstream	84.5	15.5	84	**χ^2^** = 64.307 *P* < .001
Midstream	27.7	72.3	112	
Down stream	44.2	55.8	163	
Total	48.5	51.5	359	

* *P* *<* *0·05*.

** *P* *<* *0·01*.

*** *P* *<* *0·001*.

Source: Field Survey, 2022.

The sexual desire of partners of women and climate-related risk are significantly associated (Table 8). Women shared that when there is a more climate-related risk, the sexual desire of their partners has decreased (*P* < .001). Similarly, all ethnic groups of women reported that the sexual desire of their partner decreased during the climate crisis, except Brahmin/Chhetri. Further, the upstream women have no change in sexual desire compared with women residing in midstream and downstream.

**Table 8:** Percentage of women who reported changes in sexual desire of their husbands after climate events by different characteristics.

Background characteristics	Percentage of women who reported changes in sexual desire for their partner			χ^2^ (*P* value)
	Same or increased	Decreased	Total (*N* = 359)	
Climate-related risk***				
Exposed to one or no climate-related risk	69.6	30.4	171	**χ^2^** = 66.442 *P* < .001
Exposed to at least 2 climate-related risk	26.6	73.4	188	
Age				
18–29 years	52.9	47.1	140	**χ^2^** = 3.079 *P* = 0.079
30–49	43.4	56.6	219	
Education				
Basic	45.2	54.8	168	**χ^2^** = 10.023 *P* = 0.007
Secondary and higher	61.7	38.3	81	
Nonformal and illiterate	39.1	60.9	110	
Ethnicity***				
Brahmin/Chhetri	81.9	18.1	72	**χ^2^** = 56.294 *P* < .001
Hill Janjati	36.4	63.6	121	
Hill Dalit	55.9	44.1	68	
Tharu	28.6	71.4	98	
Occupation*				
Agriculture	45.3	54.7	329	**χ^2^** = 5.043 *P* = 0.025
Other	66.7	33.3	30	
River Stream***				
Upstream	84.5	15.5	84	**χ^2^** = 71.806 *P* < .001
Midstream	24.1	75.9	112	
Down stream	43.6	56.4	163	
Total	47.1	52.9	359	

* *P* *<* *0·05*.

*** *P* *<* *0·001*.

Source: Field Survey, 2022.

### Multivariate analysis

Table 9 highlights the level of associations of the factors influencing women's experiences of utilizing sexual and reproductive health and rights. The odds of experiencing GBV were higher (aOR 3.728*) for women exposed to at least two climate-related risks. Those women who were exposed to at least two climate-related risks also showed increased workload (aOR 2.629**), intention to have more children (aOR 4.845***), knowledge on the legality of abortion (aOR 6.735***), and increased desire for sexual activity for both the individual and their partner (aOR 5.539***). The odds of GBV exposure were significantly greater for Tharu women (aOR 14.097**), however, other caste/ethnic groups have an insignificant association with GBV exposure. The odds of enhanced sexual desire were significantly higher for Hill Janjati and Tharu women (aORs over 14) and found similar patterns in their partners. The male partners whose wives were working in non-agricultural areas were less likely to be sexually active (aOR 0.352*) than their wives. Mid and downstream women had significantly reduced odds of experiencing GBV (aOR 0.015***; 0.115**) but same or increased sexual desire (aOR 8.407***) for only partners of mid-stream. Women living in the downstream had increased workloads (aOR 2.574*) but decreased odds in intention to have children, abortion knowledge, and experiencing GBV.

**Table 9:** Adjusted odds ratios on climate-related risk and gender and SRHR.

Characteristics	Adjusted odds ratio (aOR)						
	Faced GBV	Women's Autonomy	Work-load	Intension to have children	Knowledge	Sexual desire (self)	Sexual desire (partner)
Climate-related risk							
Exposed to one or no climate-related risk (ref.)							
Exposed to at least 2 climate-related risk	3.728*	1.625	2.629**	4.845***	6.735***	4.201***	5.539***
Age							
18–29 years (ref.)							
30–49	0.741	0.995	1.037	0.812	1.391	1.773	1.299
Education							
Basic (ref.)							
Secondary and higher	0.393*	1.032	1.031	1.308	1.740	0.926	0.557
Nonformal and illiterate	1.768	1.349	2.083*	0.861	0.342*	1.021	0.971
Ethnicity							
Brahmin/Chhetri (ref.)							
Hill Janjati	1.102	1.284	3.131**	0.546	1.805	14.080***	14.472***
Hill Dalit	1.620	1.367	1.286	0.680	2.746	6.022**	3.683*
Tharu	14.097**	2.558*	2.255	1.814	0.584	14.659***	13.647***
Occupation							
Agriculture (ref.)							
Other	0.596	0.842	1.308	0.355	1.864	0.534	0.352*
River Stream							
Upstream (ref.)							
Midstream	0.015 ***	2.089*	0.953	0.000	-	8.407***	10.491***
Down stream	0.115**	1.150	2.574*	0.236*	0.38***	1.892	1.880

* *P < 0·05.*

** *P < 0·01.*

*** *P < 0·001.*

Source: Field Survey, 2022.

## Discussions

Climate change poses a significant threat to the well-being of communities globally, with differential impacts on marginalized populations in developing countries. This study attempts to explore associations between climate change, gender, and sexual and reproductive health and rights in Nepal's context. The findings from this study are important to document the perceived impact on women from vulnerable communities in Nepal. The study emphasizes that climate change impacts on women vary according to their sociodemographic characteristics, including age, education, ethnicity, occupation, and residence in a vulnerable area. The study findings show that there are associations between climate change and sexual and reproductive health among women. The higher the exposure to climate-related risk, the higher is the risk of GBV. The women who are exposed to at least two climatic-related risks also show increased workload, intention to have more children, knowledge of the legality of abortion, and increased desire for sexual activity for both the individual and their partner. These factors are also affected by different socio-demographic variables such as caste/ethnicity, occupation, and river basin.

In consistent to the study, multiple studies conducted in South Asia and Nepal suggests that women are exposed more to extreme climate events such as droughts, floods, extreme rainfall, etc., and experience the differential impact of climate change due to the socio-demographical situation, the health condition, social construct, systemic gender discrimination, access to resources, decision making power, and societal expectations related to gender roles (48–50). Published literature also shows that suggests that adolescent girls and women residing in LMICs face significant SRHR challenges in the context of climate change (20). These vulnerable groups are particularly affected, highlighting the urgent need to address the unique obstacles they encounter as they navigate these pressing issues. The intersection of climate change and SRHR opens a crucial conversation about empowerment and the protection of their well-being.

The study findings also highlight increased violence among women experiencing multiple climate-related risks, indicating the interlinkages between exposure to climate-related risks and GBV. Various studies conducted across the globe have reported the interlinkages between climate change and multiple forms of violence, similar to this study, and share that the extreme effect of climate change leads to economic instability, food insecurity, worsen mental health, limit accessibility due to destroyed infrastructure, and exacerbate gender inequality, including GBV (51–56). Regardless of the high and disproportionate impact of GBV among womenduring the climate crisis, the existing plethora of evidence suggests limited intervention to prevent and respond to GBV during the climate crisis (35, 53, 56).

The study shows that womenwho faced more climate-related risks tend to show more autonomy. This seems to be linked to the fact that many of the households in the study are headed by women, mainly because many men had migrated elsewhere for work. In their absence, women took on greater responsibilities and made more decisions at home, which may explain the higher levels of autonomy observed. However, it is important to note that Nepalese society is generally patriarchal. In most cases, men are considered the heads of households, and women often have limited control over property, resources, reproductive choices, and other key decisions (57, 58). Similarly, IPCC and United Nations Framework Convention on Climate Change (UNFCCC) gender and climate change report, which suggests that women tend to have fewer assets and rights than men and are more vulnerable to losing these assets and rights due to gender roles and discrimination, structural inequalities, separation, divorce, or widowhood, and have less access to capital, extension, inputs, resources, and decision-making processes (30, 49, 59).

This study shows that women exposed to multiple climate-related risks often face an increased workload. Furthermore, the qualitative findings indicate that climate-related risks, such as droughts, floods, and landslides, have harmed marginalized people in rural Nepal, with women bearing the brunt of extensive roles, including collecting water, fodder, and firewood for cooking and drinking, participating in agricultural and livestock rearing, and providing caregiving. This heightened burden can compromise their access to health services and overall well-being. As their responsibilities grow, the time and energy available for seeking healthcare might be compromised, negatively impacting their health and limiting their ability to effectively utilize necessary health services. Multiple studies have also linked the gender impact of climate-induced disasters to adverse health consequences, including early and forced marriages, followed by early childbearing and increased workload, which leads to pelvic organ prolapse, gender-based violence (60), miscarriage, unsafe or spontaneous abortion, access to contraception (61), HIV prevalence (37), pre-term birth, low birth weight (60), and access to maternal health services including antenatal care, post-natal care, and delivery (37). Likewise, one of our study findings also shows that women face challenges in accessing abortion during climate-related risk due to disruptions in the health care services. This study also assessed knowledge of abortion legality among women who were exposed to climate-related risks. Women with non-formal or no education, residing in vulnerable geography, and downstream, have limited knowledge of abortion legality and its service availability. Therefore, it is crucial to address the intersections of climate change and abortion services to ensure that women have access to the reproductive healthcare they need, particularly in the face of the growing challenges posed by a changing climate (62, 63).

This study highlights the impact of climate change on sexual desire and fertility preference, which impacts sexual and reproductive health, which is a unique finding. On the one hand, the uncertainty and anxiety surrounding the future impacts of climate change may lead some people, particularly women, to reconsider or delay having children (37). Studies conducted in the United States found that there is an impact of climate change on fertility intentions, i.e., to have children or how many children to have, both men and women reported (64, 65). Contrary to previous studies conducted worldwide, this study reports that only a quarter of the women in our study areas want to have more children. Those who want to have more children do so in fear of losing a child due to the climate-related risk. Those are the women with higher climate-related risks. Thus, this evidence suggests that climate change may be a significant factor in shaping reproductive intentions, with potentially far-reaching implications for population dynamics and society as a whole (66). The study findings indicate that climate change has also influenced sexual desire among women, consistent with findings from other studies. Studies have shown the impact of climate change on sexual desire through direct and indirect mechanisms. Directly, extreme weather events, such as heat waves and droughts, can lead to physical discomfort and stress, which can negatively affect sexual desire and performance (7, 51, 67, 68). Indirectly, the socio-economic consequences of climate change, such as food insecurity, income loss, and migration, can lead to mental health issues, ultimately impacting sexual desire and overall well-being (51, 67, 68).

To address the disproportionate impact of climate change on gender and health, this study calls for the integration of climate change, gender, and SRHR, which requires interministerial and inter-departmental collaboration. This study also demands strengthening the healthcare ecosystems, including vulnerability assessment, training of the health workforce, orientation to community stakeholders, climate and health financing, and stockpiling of reproductive health commodities to withstand the impacts of climate change, ensuring that essential sexual and reproductive health services during times of crisis. The study findings also suggest that there is a need for consideration of the socio-demographic factors while designing the programs, whether it is at the community level or at the health system to reach to the affected population.

### Strengths and limitations of the study

This study stands out as one of the initiating efforts in Nepal that explored the complex interlinkage between climate change and its impact on women. It analyzed important but unexplored aspects such as their SRHR, gender, and autonomy, which is the strength of this study. Moreover, the findings of this study serve as evidence for development professionals, researchers, and duty bearers for the inclusion of climate, gender, and SRHR at all levels. The limitation of this study is that the study covers a particular geographical context, which limits its broader generalizability. The study only reports some of the impact of climate change on SRHR, as reported by the participants, which calls for a national-level study for a comprehensive assessment and understanding.

## Conclusion

The study concludes that women from the ethnic community, with a low level of education, residing in the vulnerable areas, like the downstream of rivers, are severely exposed to climate-related risk and experience the impact of climate change more. Increased exposure to climate-related risk has led to significant experience of gender-based violence, an intention to have more children, low SRHR-related awareness, decreased sexual desire, and increased workload among women. The impact of climate change on sexual and reproductive health was significantly associated with the number of climatic events faced, low level of education, residing downstream, and among ethnic communities. The disproportionate impact of climate change on women's sexual and reproductive health and rights in Nepal underscores the need for a resilient and gender-responsive approach to climate change adaptation. To address this, policymakers and development practitioners must understand the importance of integrating gender and SRHR in climate actions and plans. The adaptation and mitigation approach needs to include a multifaceted strategy that includes vulnerability and risk assessment, investment in climate-resilient health infrastructure, stockpiling of SRHR commodities, and enhancing the capacity of duty bearers to plan and provide SRHR services during the climate crisis and humanitarian setting. Furthermore, targeted community interventions to prevent and respond to gender-based violence, increased awareness regarding SRHR, and integrated climate program and financing into the local level planning process are key for the adaptation.

## Data Availability

The raw data supporting the conclusions of this article will be made available by the authors, without undue reservation.
